# Supplementary material for: Beyond microbial abundance: metadata integration enhances disease prediction in human microbiome studies
Source: Front Microbiol. 2026 Jan 21;16:1695501. doi: 10.3389/fmicb.2025.1695501 (PMC12869998; doi:10.3389/fmicb.2025.1695501)
Supplement: Supplementary file 1 [file Data_Sheet_1.pdf]

Supplementary Material

1 MICROBIOME STUDIES

Table S1 include references to all the studies used in this work along with their metadata. Some studies were obtained from the `curatedMetagenomicData` package (Pasolli et al., 2017).

2 MISSINGNESS THRESHOLD SENSITIVITY ANALYSIS

We performed a sensitivity analysis on the missingness threshold to assess its impact on feature retention and overall data quality. Results are shown in Figure S1. Specifically, we examined how the threshold affects the number of selected features and the *overall missing fraction* (defined as the ratio of missing data to total data). The results, shown in the plot below, reveal that both metrics remain relatively stable for thresholds up to 60%. Beyond this critical point, however, the overall missing fraction increases sharply while the number of additional features gained grows at a much slower rate. This divergence indicates that tolerating missingness above 60% yields diminishing returns: the marginal benefit of retaining a few additional features is outweighed by the substantial increase in missing data that must be imputed, potentially compromising model reliability.

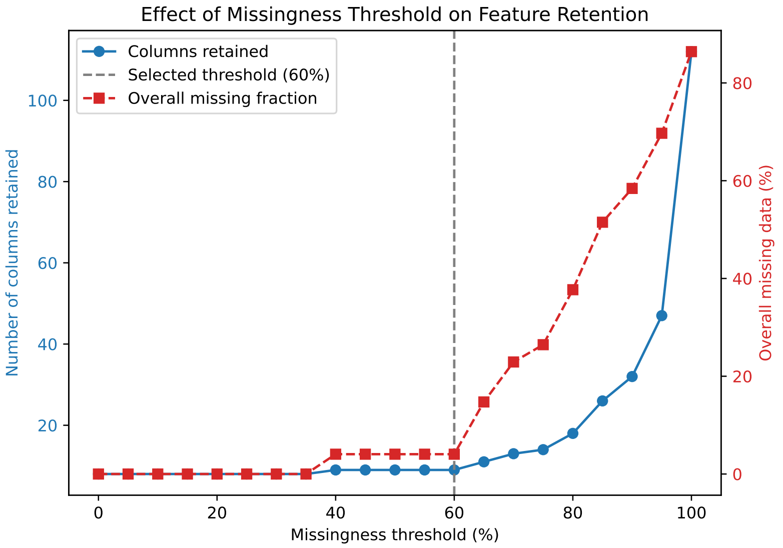

Figure S1. Sensitivity analysis of the missingness threshold. Metadata variables with less than 60% missing data were retained. The selected 60% threshold represents a trade-off between the number of retained variables and the overall fraction of missing data requiring imputation.

3 STATISTICAL COMPARISON OF JOINT (MICROBIOME+METADATA) AND MICROBIOME-ONLY MODELS

To evaluate whether microbiome+metadata (joint) training improved predictive performance compared to microbiome-only models, we conducted a paired statistical analysis for each machine learning method

and taxonomy level. For every dataset and taxonomy level, each method was trained on 30 independent train/test splits, yielding 30 AUROC values per condition (joint vs. microbiome-only).

For each dataset and level, we first compute the median AUROC over the 30 runs to obtain a single representative performance measure per condition, thereby avoiding inflation of significance due to repeated splits. The Wilcoxon signed-rank test, a non-parametric paired test, was then applied to compare the joint versus microbiome-only AUROC across datasets for each method and taxonomy level. We specifically tested the alternative hypothesis that the joint model performs better than the microbiome-only model.

To account for multiple comparisons across all method and taxonomy-level combinations, we applied the Benjamini-Hochberg procedure to control the false discovery rate (FDR). Effect sizes were quantified using Cohen's d for paired samples, calculated as the mean difference between joint and microbiome-only AUROC divided by the standard deviation of the differences. Results are reported as the mean AUROC for joint and microbiome-only models, the paired Cohen's d, and FDR-corrected p-values. Statistical significance is indicated when the corrected p-value is below 0.05, and significance levels are annotated using stars: $p < 0.05$ (*), $p < 0.01$ (**), and $p < 0.001$ (***).

4 MACHINE LEARNING MODEL HYPERPARAMETERS

Table S3 summarizes the hyperparameter values applied for each machine learning algorithm in our study. These values were the same across all taxonomic ranks.

REFERENCES

Bai, X., Narayanan, A., Skagerberg, M., Ceña-Diez, R., Giske, C. G., Strålin, K., et al. (2022). Characterization of the upper respiratory bacterial microbiome in critically Ill COVID-19 patients. *Biomedicines* 10, 982

Bedarf, J. R., Hildebrand, F., Coelho, L. P., Sunagawa, S., Bahram, M., Goeser, F., et al. (2017). Functional implications of microbial and viral gut metagenome changes in early stage L-DOPA-naïve Parkinson's disease patients. *Genome medicine* 9, 1–13

Boktor, J. C., Sharon, G., Verhagen Metman, L. A., Hall, D. A., Engen, P. A., Zreloff, Z., et al. (2023). Integrated multi-cohort analysis of the Parkinson's disease gut metagenome. *Movement Disorders* 38, 399–409

Bommana, S., Richards, G., Kama, M., Kodimerla, R., Jijakli, K., Read, T. D., et al. (2022). Metagenomic shotgun sequencing of endocervical, vaginal, and rectal samples among fijian women with and without chlamydia trachomatis reveals disparate microbial populations and function across anatomic sites: a pilot study. *Microbiology spectrum* 10, e00105–22

Boolchandani, M., Blake, K. S., Tilley, D. H., Cabada, M. M., Schwartz, D. J., Patel, S., et al. (2022). Impact of international travel and diarrhea on gut microbiome and resistome dynamics. *Nature communications* 13, 7485

Byrd, A. L., Deming, C., Cassidy, S. K., Harrison, O. J., Ng, W.-I., Conlan, S., et al. (2017). Staphylococcus aureus and staphylococcus epidermidis strain diversity underlying pediatric atopic dermatitis. *Science translational medicine* 9, eaal4651

Castro-Nallar, E., Bendall, M. L., Pérez-Losada, M., Sabuncyan, S., Severance, E. G., Dickerson, F. B., et al. (2015). Composition, taxonomy and functional diversity of the oropharynx microbiome in individuals with schizophrenia and controls. *PeerJ* 3, e1140

Chang, H.-W., Yan, D., Singh, R., Bui, A., Lee, K., Truong, A., et al. (2022). Multiomic analysis of the gut microbiome in psoriasis reveals distinct host–microbe associations. *JID Innovations* 2, 100115

Chng, K. R., Tay, A. S. L., Li, C., Ng, A. H. Q., Wang, J., Suri, B. K., et al. (2016). Whole metagenome profiling reveals skin microbiome-dependent susceptibility to atopic dermatitis flare. *Nature microbiology* 1, 1–10

David, L. A., Weil, A., Ryan, E. T., Calderwood, S. B., Harris, J. B., Chowdhury, F., et al. (2015). Gut microbial succession follows acute secretory diarrhea in humans. *MBio* 6, 10–1128

Espinoza, J. L., Harkins, D. M., Torralba, M., Gomez, A., Highlander, S. K., Jones, M. B., et al. (2018). Supragingival plaque microbiome ecology and functional potential in the context of health and disease. *MBio* 9, 10–1128

Feng, Q., Liang, S., Jia, H., Stadlmayr, A., Tang, L., Lan, Z., et al. (2015). Gut microbiome development along the colorectal adenoma–carcinoma sequence. *Nature communications* 6, 6528

Ferreiro, A. L., Choi, J., Ryou, J., Newcomer, E. P., Thompson, R., Bollinger, R. M., et al. (2023). Gut microbiome composition may be an indicator of preclinical alzheimer's disease. *Science Translational Medicine* 15, eabo2984

Franzosa, E. A., Sirota-Madi, A., Avila-Pacheco, J., Fornelos, N., Haiser, H. J., Reinker, S., et al. (2019). Gut microbiome structure and metabolic activity in inflammatory bowel disease. *Nature microbiology* 4, 293–305

Ghensi, P., Manghi, P., Zolfo, M., Armanini, F., Pasolli, E., Bolzan, M., et al. (2020). Strong oral plaque microbiome signatures for dental implant diseases identified by strain-resolution metagenomics. *npj Biofilms and Microbiomes* 6, 47

Guillén, Y., Noguera-Julian, M., Rivera, J., Casadellà, M., Zevin, A. S., Rocafort, M., et al. (2019). Low nadir cd4+ t-cell counts predict gut dysbiosis in hiv-1 infection. *Mucosal immunology* 12, 232–246

Guo, C., Che, X., Briese, T., Ranjan, A., Allicock, O., Yates, R. A., et al. (2023). Deficient butyrate-producing capacity in the gut microbiome is associated with bacterial network disturbances and fatigue symptoms in ME/CFS. *Cell host & microbe* 31, 288–304

Hall, A. B., Yassour, M., Sauk, J., Garner, A., Jiang, X., Arthur, T., et al. (2017). A novel ruminococcus gnavus clade enriched in inflammatory bowel disease patients. *Genome medicine* 9, 1–12

Hannigan, G. D., Duhaime, M. B., Ruffin IV, M. T., Koumpouras, C. C., and Schloss, P. D. (2018). Diagnostic potential and interactive dynamics of the colorectal cancer virome. *MBio* 9, 10–1128

Heintz-Buschart, A., May, P., Laczny, C. C., Lebrun, L. A., Bellora, C., Krishna, A., et al. (2016). Integrated multi-omics of the human gut microbiome in a case study of familial type 1 diabetes. *Nature microbiology* 2, 1–13

Hu, Y., Feng, Y., Wu, J., Liu, F., Zhang, Z., Hao, Y., et al. (2019). The gut microbiome signatures discriminate healthy from pulmonary tuberculosis patients. *Frontiers in cellular and infection microbiology* 9, 90

Integrative, H. (2014). The integrative human microbiome project: dynamic analysis of microbiome-host omics profiles during periods of human health and disease. *Cell host & microbe* 16, 276–289

Jie, Z., Xia, H., Zhong, S.-L., Feng, Q., Li, S., Liang, S., et al. (2017). The gut microbiome in atherosclerotic cardiovascular disease. *Nature communications* 8, 845

Jo, S., Kang, W., Hwang, Y. S., Lee, S. H., Park, K. W., Kim, M. S., et al. (2022). Oral and gut dysbiosis leads to functional alterations in parkinson's disease. *npj Parkinson's Disease* 8, 87

Karlsson, F. H., Tremaroli, V., Nookaew, I., Bergström, G., Behre, C. J., Fagerberg, B., et al. (2013). Gut metagenome in european women with normal, impaired and diabetic glucose control. *Nature* 498, 99–103

Khachatryan, L., Xiang, Y., Ivanov, A., Glaab, E., Graham, G., Granata, I., et al. (2023). Results and lessons learned from the sbv IMPROVER metagenomics diagnostics for inflammatory bowel disease challenge. *Scientific Reports* 13, 6303

Kieser, S., Sarker, S. A., Sakwinska, O., Foata, F., Sultana, S., Khan, Z., et al. (2018). Bangladeshi children with acute diarrhoea show faecal microbiomes with increased streptococcus abundance, irrespective of diarrhoea aetiology. *Environmental microbiology* 20, 2256–2269

Laske, C., Müller, S., Preische, O., Ruschil, V., Munk, M. H., Honold, I., et al. (2022). Signature of alzheimer's disease in intestinal microbiome: results from the alzbiom study. *Frontiers in Neuroscience* 16, 792996

Li, J., Zhao, F., Wang, Y., Chen, J., Tao, J., Tian, G., et al. (2017). Gut microbiota dysbiosis contributes to the development of hypertension. *Microbiome* 5, 1–19

Liu, Q., Mak, J. W. Y., Su, Q., Yeoh, Y. K., Lui, G. C.-Y., Ng, S. S. S., et al. (2022). Gut microbiota dynamics in a prospective cohort of patients with post-acute covid-19 syndrome. *Gut* 71, 544–552

Loomba, R., Seguritan, V., Li, W., Long, T., Klitgord, N., Bhatt, A., et al. (2019). Gut microbiome-based metagenomic signature for non-invasive detection of advanced fibrosis in human nonalcoholic fatty liver disease. *Cell metabolism* 30, 607

Maya-Lucas, O., Murugesan, S., Nirmalkar, K., Alcaraz, L. D., Hoyo-Vadillo, C., Pizano-Zárate, M. L., et al. (2019). The gut microbiome of mexican children affected by obesity. *Anaerobe* 55, 11–23

McDonald, D., Hyde, E., Debelius, J. W., Morton, J. T., Gonzalez, A., Ackermann, G., et al. (2018). American gut: an open platform for citizen science microbiome research. *Msystems* 3, 10–1128

Nagata, N., Nishijima, S., Kojima, Y., Hisada, Y., Imbe, K., Miyoshi-Akiyama, T., et al. (2022). Metagenomic identification of microbial signatures predicting pancreatic cancer from a multinational study. *Gastroenterology* 163, 222–238

Nagy-Szakal, D., Williams, B. L., Mishra, N., Che, X., Lee, B., Bateman, L., et al. (2017). Fecal metagenomic profiles in subgroups of patients with myalgic encephalomyelitis/chronic fatigue syndrome. *Microbiome* 5, 1–17

Pasolli, E., Schiffer, L., Manghi, P., Renson, A., Obenchain, V., Truong, D. T., et al. (2017). Accessible, curated metagenomic data through experimenthub. *Nature methods* 14, 1023–1024

Pienkowska, K., Pust, M.-M., Gessner, M., Gaedcke, S., Thavarasa, A., Rosenboom, I., et al. (2023). The cystic fibrosis upper and lower airway metagenome. *Microbiology Spectrum* 11, e03633–22

Polster, S. P., Sharma, A., Tanes, C., Tang, A. T., Mericko, P., Cao, Y., et al. (2020). Permissive microbiome characterizes human subjects with a neurovascular disease cavernous angioma. *Nature communications* 11, 2659

Pust, M.-M., Wiehlmann, L., Davenport, C., Rudolf, I., Dittrich, A.-M., and Tümmler, B. (2020). The human respiratory tract microbial community structures in healthy and cystic fibrosis infants. *npj Biofilms and Microbiomes* 6, 61

Qi, X., Yun, C., Sun, L., Xia, J., Wu, Q., Wang, Y., et al. (2019). Gut microbiota–bile acid–interleukin-22 axis orchestrates polycystic ovary syndrome. *Nature medicine* 25, 1225–1233

Qian, Y., Yang, X., Xu, S., Huang, P., Li, B., Du, J., et al. (2020). Gut metagenomics-derived genes as potential biomarkers of parkinson's disease. *Brain* 143, 2474–2489

Qin, N., Yang, F., Li, A., Prifti, E., Chen, Y., Shao, L., et al. (2014). Alterations of the human gut microbiome in liver cirrhosis. *Nature* 513, 59–64

Rosa, B. A., Supali, T., Gankpala, L., Djuardi, Y., Sartono, E., Zhou, Y., et al. (2018). Differential human gut microbiome assemblages during soil-transmitted helminth infections in indonesia and liberia. *Microbiome* 6, 1–19

Rubel, M. A., Abbas, A., Taylor, L. J., Connell, A., Tanes, C., Bittinger, K., et al. (2020). Lifestyle and the presence of helminths is associated with gut microbiome composition in cameroonians. *Genome biology* 21, 1–32

Sankaranarayanan, K., Ozga, A. T., Warinner, C., Tito, R. Y., Obregon-Tito, A. J., Xu, J., et al. (2015). Gut microbiome diversity among cheyenne and arapaho individuals from western oklahoma. *Current Biology* 25, 3161–3169

Sun, H., Guo, Y., Wang, H., Yin, A., Hu, J., Yuan, T., et al. (2023). Gut commensal Parabacteroides distasonis alleviates inflammatory arthritis. *Gut* 72, 1664–1677

Sun, Z., Zhang, M., Li, M., Bhaskar, Y., Zhao, J., Ji, Y., et al. (2022). Interactions between human gut microbiome dynamics and sub-optimal health symptoms during seafaring expeditions. *Microbiology Spectrum* 10, e00925–21

Tay, A. S., Li, C., Nandi, T., Chng, K. R., Andiappan, A. K., Mettu, V. S., et al. (2021). Atopic dermatitis microbiomes stratify into ecologic dermotypes enabling microbial virulence and disease severity. *Journal of Allergy and Clinical Immunology* , 1329–1340

Ventura, R., Iizumi, T., Battaglia, T., Liu, M., Perez-Perez, G., Herbert, J., et al. (2019). Gut microbiome of treatment-naïve MS patients of different ethnicities early in disease course. *Scientific reports* 9, 16396

Vincent, C., Miller, M. A., Edens, T. J., Mehrotra, S., Dewar, K., and Manges, A. R. (2016). Bloom and bust: intestinal microbiota dynamics in response to hospital exposures and clostridium difficile colonization or infection. *Microbiome* 4, 1–11

Wallen, Z. D., Demirkan, A., Twa, G., Cohen, G., Dean, M. N., Standaert, D. G., et al. (2022). Metagenomics of parkinson's disease implicates the gut microbiome in multiple disease mechanisms. *Nature communications* 13, 6958

Wang, Z., Liang, L., Liu, L., Wang, Z., Wang, Y., Yu, Z., et al. (2023). Changes in the Gut Microbiome Associated with Intussusception in Patients with Peutz-Jeghers Syndrome. *Microbiology Spectrum* 11, e02819–22

Weng, Y. J., Gan, H. Y., Li, X., Huang, Y., Li, Z. C., Deng, H. M., et al. (2019). Correlation of diet, microbiota and metabolite networks in inflammatory bowel disease. *Journal of Digestive Diseases* 20, 447–459

Xiong, R., Gunter, C., Fleming, E., Vernon, S. D., Bateman, L., Unutmaz, D., et al. (2023). Multi-omics of gut microbiome-host interactions in short- and long-term myalgic encephalomyelitis/chronic fatigue syndrome patients. *Cell Host & Microbe* 31, 273–287.e5

Xiong, Z., Peng, K., Song, S., Zhu, Y., Gu, J., Huang, C., et al. (2022). Cerebral intraparenchymal hemorrhage changes patients' gut bacteria composition and function. *Frontiers in Cellular and Infection Microbiology* 12, 829491

Yachida, S., Mizutani, S., Shiroma, H., Shiba, S., Nakajima, T., Sakamoto, T., et al. (2019). Metagenomic and metabolomic analyses reveal distinct stage-specific phenotypes of the gut microbiota in colorectal cancer. *Nature medicine* 25, 968–976

Yan, Q., Gu, Y., Li, X., Yang, W., Jia, L., Chen, C., et al. (2017). Alterations of the gut microbiome in hypertension. *Frontiers in cellular and infection microbiology* 7, 381

Ye, Z., Zhang, N., Wu, C., Zhang, X., Wang, Q., Huang, X., et al. (2018). A metagenomic study of the gut microbiome in behcet's disease. *Microbiome* 6, 1–13

Yu, J., Feng, Q., Wong, S. H., Zhang, D., yi Liang, Q., Qin, Y., et al. (2017). Metagenomic analysis of faecal microbiome as a tool towards targeted non-invasive biomarkers for colorectal cancer. *Gut* 66, 70–78

Zeller, G., Tap, J., Voigt, A. Y., Sunagawa, S., Kultima, J. R., Costea, P. I., et al. (2014). Potential of fecal microbiota for early-stage detection of colorectal cancer. *Molecular Systems Biology* 10, 766

Zhang, F., Wan, Y., Zuo, T., Yeoh, Y. K., Liu, Q., Zhang, L., et al. (2022). Prolonged impairment of short-chain fatty acid and l-isoleucine biosynthesis in gut microbiome in patients with covid-19. *Gastroenterology* 162, 548–561

Zhou, T., Wu, J., Zeng, Y., Li, J., Yan, J., Meng, W., et al. (2022). Sars-cov-2 triggered oxidative stress and abnormal energy metabolism in gut microbiota. *MedComm* 3, e112

Zhu, F., Ju, Y., Wang, W., Wang, Q., Guo, R., Ma, Q., et al. (2020). Metagenome-wide association of gut microbiome features for schizophrenia. *Nature communications* 11, 1612

Zhu, Q., Hou, Q., Huang, S., Ou, Q., Huo, D., Vázquez-Baeza, Y., et al. (2021). Compositional and genetic alterations in graves' disease gut microbiome reveal specific diagnostic biomarkers. *The ISME journal* 15, 3399–3411

Zinkernagel, M. S., Zysset-Burri, D. C., Keller, I., Berger, L. E., Leichtle, A. B., Largiadèr, C. R., et al. (2017). Association of the intestinal microbiome with the development of neovascular age-related macular degeneration. *Scientific reports* 7, 40826

Zuo, T., Zhang, F., Lui, G. C., Yeoh, Y. K., Li, A. Y., Zhan, H., et al. (2020). Alterations in gut microbiota of patients with covid-19 during time of hospitalization. *Gastroenterology* 159, 944–955

Zuo, W., Wang, B., Bai, X., Luan, Y., Fan, Y., Michail, S., et al. (2022). 16s rrna and metagenomic shotgun sequencing data revealed consistent patterns of gut microbiome signature in pediatric ulcerative colitis. *Scientific Reports* 12, 6421

Zysset-Burri, D. C., Keller, I., Berger, L. E., Largiadèr, C. R., Wittwer, M., Wolf, S., et al. (2020). Associations of the intestinal microbiome with the complement system in neovascular age-related macular degeneration. *NPJ genomic medicine* 5, 34

Project	Reference	Sample Size	% of Missing	Disease Category
2022_American	(McDonald et al., 2018)	648	21%	*Multiple Diseases
2017_Zinkernagel	(Zinkernagel et al., 2017)	23	33%	AMD
2020_Zysset-Burri-AMD	(Zysset-Burri et al., 2020)	90	33%	
2021_Zhu	(Zhu et al., 2021)	148	11%	Autoimmune
2018_YeZ	(Ye et al., 2018)	64	11%	
2022_Nagata	(Nagata et al., 2022)	560	44%	Cancer
2015_FengQ	(Feng et al., 2015)	154	22%	
2019_YachidaS	(Yachida et al., 2019)	614	22%	
2014_Zeller	(Zeller et al., 2014)	193	11%	
2017_YuJ	(Yu et al., 2017)	128	22%	
2018_HanniganGD	(Hannigan et al., 2018)	52	11%	
2017_JieZ	(Jie et al., 2017)	385	11%	Cardio
2017_Yan	(Yan et al., 2017)	116	11%	Cardio
2022_Xiong	(Xiong et al., 2022)	20	0%	*Cerebral*
2020_Polster	(Polster et al., 2020)	154	33%	*Brain*
2017_LiJ	(Li et al., 2017)	196	33%	Cardio
2020_Zysset-Burri-Cardio	(Zysset-Burri et al., 2020)	59	33%	Cardio
2014_Qin	(Qin et al., 2014)	222	0%	Liver Disorders
2019_LoombaR	(Loomba et al., 2019)	86	44%	
2021_Tay	(Tay et al., 2021)	90	11%	Dermatologic
2016_ChngKR	(Chng et al., 2016)	68	33%	
2022_Chang	(Chang et al., 2022)	48	0%	
2017_Byrd	(Byrd et al., 2017)	232	33%	
2015_SankaranarayananK	(Sankaranarayanan et al., 2015)	36	11%	Diabetes
2013_KarlssonFH	(Karlsson et al., 2013)	144	11%	
2016_Heintz-BuschartA	(Heintz-Buschart et al., 2016)	53	11%	
2019_Qi	(Qi et al., 2019)	93	22%	Endocrine Disorder
2019_Maya-Lucas	(Maya-Lucas et al., 2019)	20	22%	
2015_DavidLA	(David et al., 2015)	22	33%	Gastrointestinal
2019_Franzosa	(Franzosa et al., 2019)	213	22%	
2022_IHMP	(Integrative, 2014)	1,258	22%	
2020_RubelMA	(Rubel et al., 2020)	175	22%	
2016_VincentC	(Vincent et al., 2016)	170	11%	
2022_Zuo	(Zuo et al., 2022)	35	11%	
2017_HallAB	(Hall et al., 2017)	236	44%	
2018_KieserS	(Kieser et al., 2018)	27	44%	
2018_RosaBA	(Rosa et al., 2018)	24	44%	
2019_Weng	(Weng et al., 2019)	79	33%	
2022_Boolchandani	(Boolchandani et al., 2022)	350	11%	
2022_Sun	(Sun et al., 2022)	154	33%	
2023_Khachatryan	(Khachatryan et al., 2023)	105	33%	
2023_Wang	(Wang et al., 2023)	88	33%	
2022_Bommana	(Bommana et al., 2022)	20	16%	Genitourinary
2019_Guillen	(Guillén et al., 2019)	156	0%	Immune
2023_Sun	(Sun et al., 2023)	47	44%	Musculoskeletal
2023_Guo	(Guo et al., 2023)	197	11%	Neurological
2023_Xiong	(Xiong et al., 2023)	238	0%	
2023_Ferreiro	(Ferreiro et al., 2023)	163	11%	
2023_Boktor	(Boktor et al., 2023)	215	11%	
2015_Castro-NallarE	(Castro-Nallar et al., 2015)	32	22%	
2020_ZhuF	(Zhu et al., 2020)	171	11%	
2022_Jo	(Jo et al., 2022)	154	22%	
2019_Ventura	(Ventura et al., 2019)	48	11%	
2022_Wallen	(Wallen et al., 2022)	723	33%	
2017_Bedarf	(Bedarf et al., 2017)	58	11%	
2020_Qian	(Qian et al., 2020)	80	33%	
2017_NagySzakalD	(Nagy-Szakal et al., 2017)	100	16%	
2022_Laske	(Laske et al., 2022)	175	11%	
2019_GhensiP	(Ghensi et al., 2020)	60	55%	Oral
2018_Espinoza	(Espinoza et al., 2018)	83	22%	
2022_Zhou	(Zhou et al., 2022)	41	11%	Pulmonary
2019_Hu	(Hu et al., 2019)	77	11%	
2022_Bai	(Bai et al., 2022)	55	33%	
2020_Pust	(Pust et al., 2020)	97	44%	
2022_Liu	(Liu et al., 2022)	110	33%	
2023_Pienkowska	(Pienkowska et al., 2023)	200	46%	
2022_Zhang	(Zhang et al., 2022)	207	33%	
2020_Zuo	(Zuo et al., 2020)	69	33%	

Table S1. Microbiome studies used our meta-analysis work. We used a total of 11,208 microbiome profiles. Percentage of missin data is based on the nine metadata variables retained. *The American Gut Project was a crowdsourced study with diverse participants having various health conditions rather than focusing on one specific disease.

Level	Method	$\text{Joint}_{\text{mean}}$	$\text{Microb}_{\text{mean}}$	Cohen's d	p-value	p-value corr	Significant	Stars
Kingdom	KNN	0.633	0.609	0.444	0.000131	0.001830	True	**
Kingdom	LC	0.614	0.584	0.334	0.026939	0.034286	True	*
Kingdom	LinearSVC	0.642	0.598	0.373	0.010201	0.016801	True	*
Kingdom	RF	0.689	0.644	0.474	0.000008	0.000218	True	***
Phylum	KNN	0.652	0.646	0.332	0.007138	0.013324	True	*
Phylum	LC	0.640	0.627	0.302	0.006230	0.013324	True	*
Phylum	LinearSVC	0.665	0.649	0.238	0.135795	0.152090	False	
Phylum	RF	0.729	0.703	0.359	0.000397	0.003709	True	**
Class	KNN	0.662	0.657	0.321	0.001244	0.006967	True	**
Class	LC	0.650	0.643	0.250	0.014365	0.021083	True	*
Class	LinearSVC	0.677	0.664	0.247	0.011776	0.018318	True	*
Class	RF	0.734	0.712	0.313	0.004853	0.012509	True	*
Order	KNN	0.693	0.688	0.358	0.002768	0.008610	True	**
Order	LC	0.672	0.665	0.318	0.000548	0.003833	True	**
Order	LinearSVC	0.712	0.699	0.331	0.007741	0.013547	True	*
Order	RF	0.739	0.720	0.305	0.006725	0.013324	True	*
Family	KNN	0.703	0.702	0.138	0.125041	0.145881	False	
Family	LC	0.688	0.684	0.321	0.001749	0.008161	True	**
Family	LinearSVC	0.740	0.736	0.134	0.253571	0.273076	False	
Family	RF	0.736	0.723	0.283	0.002329	0.008610	True	**
Genus	KNN	0.710	0.709	0.248	0.045329	0.055183	False	
Genus	LC	0.699	0.698	0.330	0.004914	0.012509	True	*
Genus	LinearSVC	0.757	0.752	0.284	0.002720	0.008610	True	**
Genus	RF	0.740	0.729	0.295	0.005911	0.013324	True	*
Species	KNN	0.718	0.717	0.352	0.015793	0.021083	True	*
Species	LC	0.705	0.704	0.222	0.015812	0.021083	True	*
Species	LinearSVC	0.763	0.763	-0.033	0.418899	0.418899	False	
Species	RF	0.757	0.755	0.095	0.409822	0.418899	False	

Table S2. Mean AUROC for joint (microbiome+metadata) versus microbiome-only models across taxonomy levels and ML methods. Statistical hypothesis tests (Wilcoxon signed-rank, FDR-corrected) were performed to compare joint and microbiome-only performance, with effect sizes reported as Cohen's d and significance indicated by stars ($p < 0.05$, *$p < 0.01$, **$p < 0.001$). Overall, joint models tend to outperform microbiome-only models, especially at higher taxonomic levels.

Model	Hyperparameters
Logistic Regression	$C \in \{0.01, 0.1, \mathbf{1.0}, 10, 100\}$
K-NN	$k \in \{3, \mathbf{5}, 7, 9, 11\}$
Linear SVC	$C \in \{0.01,\ \mathbf{0.1}, 1.0, 10, 100\}$
Random Forest	n_estimators $\in \{50, \mathbf{100}, 200\}$, max_depth $\in \{\mathbf{5}, 10, 20\}$

Table S3. Hyperparameter values evaluated for each machine learning model via cross-validation. Optimal hyperparameters (highlighted in bold) were selected based on validation set performance and used for final test set evaluation.
